# Monitoring climate change and child health: The case for putting children in all policies

**DOI:** 10.1111/jpc.15757

**Published:** 2021-11-18

**Authors:** Marina Romanello, Alice McGushin, Frances A S MacGuire, Peter D Sly, Bethany Jennings, Jennifer Requejo, Anthony Costello

**Affiliations:** ^1^ Institute for Global Health University College London London United Kingdom; ^2^ Children's Health and Environment Program, Child Health Research Centre The University of Queensland Brisbane Queensland Australia; ^3^ Division of Data, Analytics, Planning and Monitoring United Nations Children's Fund New York New York United States

## Abstract

Climate change is threatening the health of current and future generations of children. The most recent evidence from the *Lancet* Countdown: Tracking Progress on Health and Climate Change finds declining trends in yield potential of major crops, rising heatwave exposures, and increasing climate suitability for the transmission of infectious diseases, putting at risk the health and wellbeing of children around the world. However, if children are considered at the core of planning and implementation, the policy responses to climate change could yield enormous benefits for the health and wellbeing of children throughout their lives. Child health professionals have a role to play in ensuring this, with the beneficiaries of their involvement ranging from the individual child to the global community. The newly established Children in All Policies 2030 initiative will work with the *Lancet* Countdown to provide the evidence on the climate change responses necessary to protect and promote the health of children.

While the last three decades saw large improvements in child health and wellbeing, advancement towards the Sustainable Development Goals has been slow,^1^ major disparities exist between and within countries and the COVID‐19 pandemic has further hampered progress.

Climate change is further adding to the risks, threatening the lives of children around the world – and if unabated, it could undermine decades of gains in global health.^2^ The world is already 1.2°C warmer than it was in the pre‐industrial period. Unless the global response rapidly accelerates towards decarbonisation, it is set to warm to 3°C or more by the end of the century.^3^ A new study shows the planet is trapping roughly double the amount of heat in the atmosphere than it did nearly 15 years ago, far more rapidly than expected.^4^

Children are particularly vulnerable to the impacts of climate change.^5^ Harmful exposures during childhood can irreversibly define physical and mental health and wellbeing, and children's reliance on adults for their physical safety and emotional development limits their capacity to control their environment. Through increased exposure to extreme weather events, increased environmental suitability for infectious disease transmission and threats to food and water security, climate change will directly impact on children's health and add extra pressure on already overwhelmed health systems, further undermining the rights of the child to good health and access to health care, to an adequate standard of living and to social security.^6^ The impacts of climate change and subsequent inequalities will be felt most by the world's poorest children. Children born today will inherit a warming world, facing increasing impacts of climate change throughout their lifetimes.

*The Lancet Countdown: Tracking Progress on Health and Climate Change* is an independent, international, multi‐disciplinary collaboration that monitors, through annually updated indicators, the health dimensions of climate change.^7^ Year on year, its data expose the compounded multidimensional health impacts of climate change and reveal the risks it poses to children's surviving, health and wellbeing – as well as the health opportunities of an accelerated response.^8^

This paper presents key findings from the *Lancet* Countdown, including evidence on the potential to improve children's health through climate change action, and key considerations for a way forward – including the role of the health professionals in safeguarding children's futures from this rising risk.

## Quantifying Threats of Climate Change to children's Flourishing: Key Findings from the *Lancet* Countdown

### Food security

Good nutrition is the cornerstone of children's health and development. Yet, in 2020, 149.2 million children under 5 years of age globally were defined as stunted and 45.4 million as wasted.^9^ While progress in improving children's nutrition has been made over the past 20 years, climate change is already affecting food security in many places of the world.^10^

**Fig. 1:**
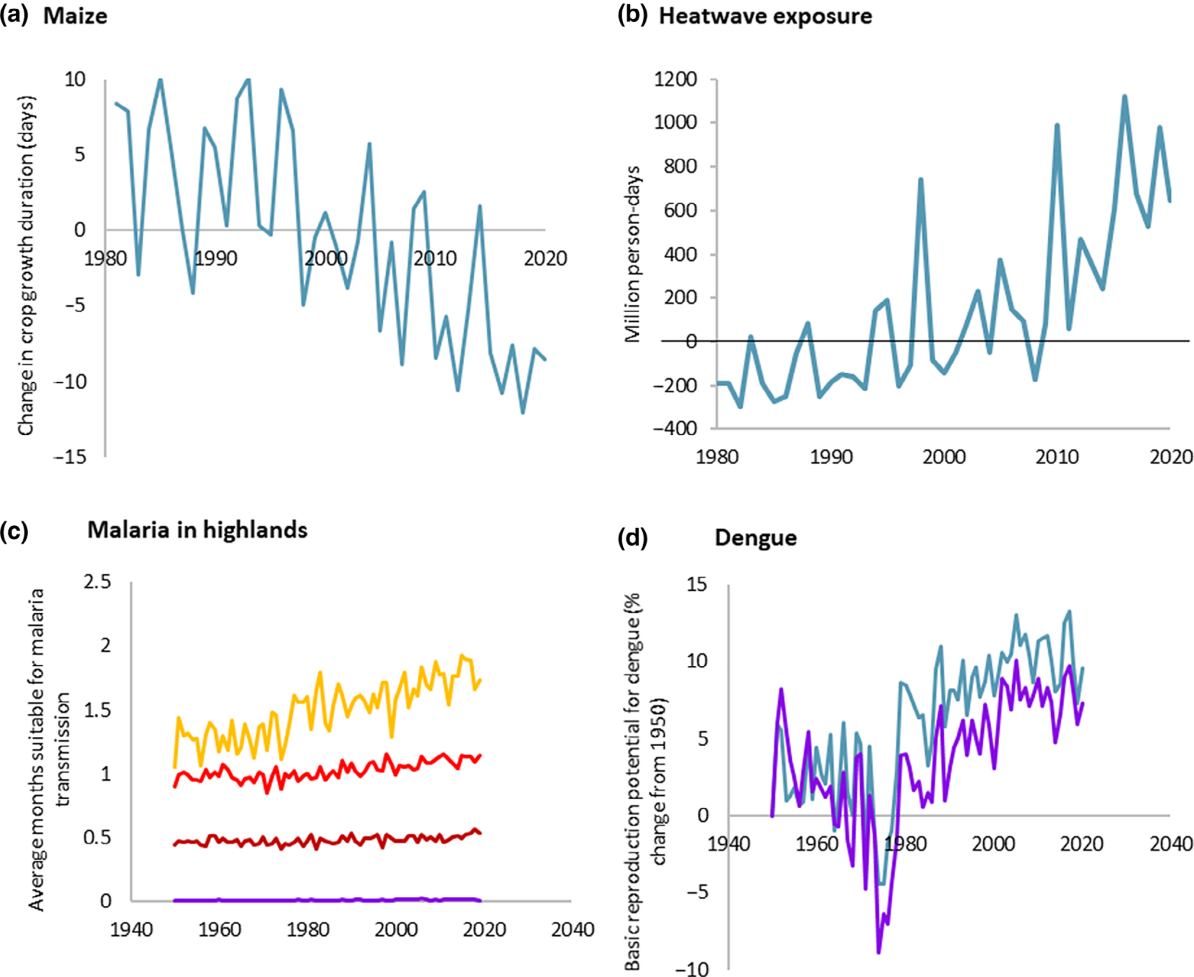
Climate change‐related health risks: (a) Crop yield potential for maize, as measured through crop growth duration; (b) change in person‐days of heatwave exposure of infants under 1 year of age, relative to a 1986–2005 baseline; (c) number of months suitable for malaria transmission in highland areas as a result of climatological changes, per HDI country group: (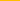) low HDI; (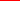) medium HDI; (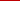) high HDI; (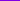) very high HDI; (d) change in the basic reproduction potential for dengue transmission as a result of climatological changes, by Aedes vector (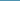) *Aedes aegypti* and (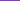) *A. albopictus* (adapted from Romanello *et al*., ^7^ with permission).

As a result of rising temperatures, the data from the *Lancet* Countdown show that the crop yield potential for maize, winter wheat, soybean and rice has decreased globally by 6.0%, 3.0%, 5.4%, and 1.8% respectively since 1981–2010 (Indicator 1.4.1: Terrestrial Food Security and Undernutrition) (Fig. 1a).^7^ Moreover, the increased frequency and intensity of drought events further add to the food insecurity risks. Indeed, the 5 years with the most global land area affected by extreme drought have all occurred since 2015 (Indicator 1.2.2: Drought).^7^

### Heat exposure

Exposure of young children to extremes of heat can lead to electrolyte imbalance, kidney disease and exacerbation of asthma.^11^ With climate change causing more frequent and intense extreme temperatures, such exposure is on the rise: the *Lancet* Countdown estimates that there were 645 million additional person‐days of heatwave exposure among children under 1‐year old in 2020 compared to the average for 1986–2005 – equivalent to 4.6 additional days of exposure per infant (Indicator 1.1.2: Exposure of Vulnerable Populations to Heatwaves, Fig. 1b).^7^ As these trends continue, young children will be under increasing risk from heat exposure – and targeted adaptation is critical to protect their health.

### Infectious diseases

The changing environmental conditions are increasing the risk of transmission of many infectious diseases. Coastal areas with environmentally suitable conditions for *Vibrio cholerae* transmission, for example, have increased significantly in the African and Eastern Mediterranean regions between 2003 and 2019, putting children in locations with reduced sanitation at risk (Indicator 1.3.1: Climate Suitability for Infectious Disease Transmission).^7^ Similarly, the changing climatic conditions are also changing the risk of transmission of vector‐borne diseases. Such is the case for malaria, one of the three leading causes for infectious disease‐related mortality in children, and which affects children under 5 years more than any other age group.^12^ The *Lancet* Countdown estimates that, as a result of changes in precipitation, temperature and humidity, the length of transmission season for *Plasmodium falciparum* malaria had increased by 39% and 15% in 2020 with respect to a 1950–1959 highlands of countries scoring low and medium on the United Nations (UN) Human Development Index respectively (Fig. 1c). The basic reproduction potential for dengue has also increased rapidly from the 1950s, and, in 2020, was 10% higher for the transmission by *Aedes aegypti* and 7% higher for *A. albopictus* (Indicator 1.3.1: Climate Suitability for Infectious Disease Transmission) (Fig. 1d).^7^ These changes are putting eradication efforts at risk, and the increased incidence of these diseases adds extra pressure to frequently overwhelmed health systems.

## The Importance of Considering Children in All Policies

As the previous section highlighted, climate change threatens children's health and wellbeing. However, prioritising children in climate policies has the potential to transform every dimension of their health and wellbeing for the better, by maximising their multiple health co‐benefits.

### Mitigation, cleaner air and safer streets

Exposure to air pollution is particularly harmful to children, with outcomes including reduced lung function, asthma, preterm birth, low birthweight, neurodevelopmental impairment, increased paediatric cancer incidence and increased incidence of chronic diseases in adulthood.^13^ Today, 91% of the world's population still live in places where the level of air pollution exceed limits recommended by the WHO.^14^ The *Lancet* Countdown estimates that chronic exposure to the air concentrations of fine particulate matter pollution (PM_2.5_) observed in 2019 would lead to 3.3 million avoidable deaths annually – a one‐third of which are attributable to the combustion of fossil fuels (Indicator 3.3: Mortality from Ambient Air Pollution by Sector).^7^

A major contributor to both air pollution and greenhouse gas emissions is road transport, for which 95.5% of the energy still comes from fossil fuel burning (Indicator 3.4: Sustainable and Healthy Road Transport).^7^ Urban redesign policies that promote the uptake of active forms of travel (walking, cycling) can deliver not only cleaner air, but also safer and more sociable cities in which children can thrive and enjoy increased physical activity and mobility, reduced traffic‐related noise pollution and reduced road injuries.^13^, ^15^ Considering road injuries were the leading cause of death in the 10–14 and 15–19 age groups globally and third leading cause of death in the 5–9 age group in 2016,^16^ moving to active and sustainable transport could deliver multidimensional benefits for children's health.

### Green space

Green‐based solutions, including increases in urban green space and tree canopy coverage, are key to climate change mitigation, both through CO_2_ absorption and by reducing urban temperature (and thus air conditioning use). Urban green spaces also benefit overall health, lowering mortality risk, and improving mental health.^17^ Fundamental to children's physical and mental development, urban green spaces also provide opportunities for physical activity, play and recreation.^18^ The *Lancet* Countdown's latest report shows that, in 2020, only 27% of urban centres were classified as being moderately green or above (Indicator 2.3.3: Urban Green Space) .^7^ This level of greenness varied between 17% of urban centres in low Human Development Index countries and 39% of urban centres in very high Human Development Index countries, exposing global inequalities and the need for accelerated intervention.

### Employment opportunities in a green economy

A rapid transition towards a low‐carbon economy is central to climate change mitigation. The associated employment opportunities will shape the future socio‐economic conditions of today's children – a major determinant of health and wellbeing.^19^ While some jobs in carbon‐intensive industries will be lost, the transition to a green economy is projected to result in net job generation.^20^ Data from the *Lancet* Countdown suggest that this transition has already begun: renewable energy provided 11.5 million jobs in 2019, a 4.2% rise from 2018; while direct employment in fossil fuel extraction declined by 14% from 2019 to 2020 (Indicator 4.2.2: Employment in Low‐Carbon and High‐Carbon Industries).^7^ However, careful policy‐making, institutional involvement and effective implementation are essential to ensure a just transition, with equitable skill development and future opportunities for all children.^20^

## The Way Forward

Sound policymaking to protect children from climate change impacts must be underpinned by evidence. The *Lancet* Countdown's indicators support this, offering data on some of the main risks that climate change poses on children's health. However, further work is needed to quantify how these are impacting on children's health specifically, and to capture other climate change‐related impacts of particular importance to them, such as those on mental health and migration. This task is complicated by the lack of standardised data at a global scale: while large amounts of data on child health and wellbeing are collected around the world, these are often not comparable between different locations.^8^ Moreover, even in cases where child health and development indicators exist, attributing observed changes in health to climate change risks still remains a challenge. Better data collection, integration and synthesis are therefore necessary to inform decision‐making that protects children's futures.

As policies for climate change adaptation and mitigation are designed and implemented, putting children at their centre will help maximise the benefits to their health and wellbeing, as described above. Incorporating the voice of children and young people in environment and climate‐related decision‐making processes is critical to ensure that their needs are met and their rights upheld. The importance of this was recognised in the 1992 Rio Declaration.^21^ Yet, 30 years on, children and young people are still frustrated that not enough has been done to act on climate change, and that their voices and their futures are not being adequately considered – and they are calling for action. Millions of children and young people were mobilised through the School Strikes for Climate movement of 2019, demanding adults to protect their futures. Greta Thunberg is renowned for raising these demands, and for highlighting the urgency of the climate crisis from a young person's perspective. Many other young persons have emerged over the past few years as influential activists, including the First Nation Canadian teenager, Autumn Peltier (who campaigns for clean water for Indigenous communities) and Lesein Mutunkei (a Kenyan high school student who has creatively raised awareness about deforestation). These voices must be listened to, and their goals supported.

### The role of child health professionals

As presented in a declaration released by the International Society for Social Paediatrics and Child Health in March 2021,^22^ child health professionals have a key role to play in promoting the way forward. By providing the knowledge and evidence to prioritise children's health, they can help ensure the child health co‐benefits of mitigation actions are maximised, and that adaptation to climate change protects child health both within clinical practice and in other health‐relevant areas (disaster preparedness; sanitation and hygiene; food systems; water security; energy access and the built environment). They can also raise the voice of children and young people, working with and supporting advocacy efforts in relevant fora, and ensuring that they are meaningfully involved in policy processes. Moreover, as authoritative voices on the subject, child health professionals can contribute to educating and building awareness on climate change and health, and through data collection and research, they can help bridge the gaps in data and evidence. Importantly, all health professionals also have a role to play in climate change mitigation itself: the health‐care sector is responsible for around 5% of total global emissions (Indicator 3.6: Healthcare Sector Emissions).^7^ To keep their oath of ‘doing no harm’, they must promote decarbonisation of their own practice.

To make this possible, the education of child health professionals at all levels of training is essential. Leading examples of this are already happening around the world, from a presentation at the annual congress of the Union of Paediatricians of Russia,^23^ to a news article from the Nicaraguan Paediatric Society,^24^ and a 1‐day course run by the Argentinian Paediatric Society.^25^ But much work still remains to ensure climate change is streamlined into medical curricula.

## Conclusion

The evidence is incontrovertible: climate change is already impacting on the health of children around the world and undermining their futures. Investing in children and young people is key to the success of every society, with benefits to communities and economies.^8^ Acting to mitigate climate change is critical, and countries must adapt to its unavoidable impacts. Encouraging Governments to view every policy developed through a ‘child focused’ lens that considers the implications of each policy for child health and wellbeing would deliver enormous dividends on children's health and wellbeing, with intergenerational benefits.

To address these challenges, Children in All Policies 2030 (CAP‐2030) was launched in April 2021. This initiative aims at joining the voices of ‘children and young people, activists, civil society institutions, religious groups, UN organisations, politicians, governments, private sector leaders, academics, and others working to centre children's health and wellbeing in the urgent work of sustainable development’. By joining forces with the *Lancet* Countdown on Climate Change, this new initiative builds on the ‘A future for the world's children? A WHO‐UNICEF‐Lancet Commission’ report published in 2020,^8^ to implement its recommendations, and drive action on climate change. It will do this by amplifying the voices and perspectives of children and by supporting policymakers with evidence and data about the impact on children, and what works to protect them now and in the future.

As the world recovers from the COVID‐19 pandemic, it is getting dangerously close to missing the goal of keeping global warming to under 1.5°C. The time to act is now. Child health professionals are essential in protecting the health of present and future generations, and ensuring this happens in the only way possible: by putting children at the centre of all policies.
